# ADP Ribosylation Factor 6 Regulates Neuronal Migration in the Developing Cerebral Cortex through FIP3/Arfophilin-1-dependent Endosomal Trafficking of N-cadherin

**DOI:** 10.1523/ENEURO.0148-16.2016

**Published:** 2016-08-29

**Authors:** Yoshinobu Hara, Masahiro Fukaya, Kanehiro Hayashi, Takeshi Kawauchi, Kazunori Nakajima, Hiroyuki Sakagami

**Affiliations:** 1 Department of Anatomy, Kitasato University School of Medicine, Sagamihara, Kanagawa 252-0374, Japan; 2 Department of Anatomy, Keio University School of Medicine, Tokyo 160-8582, Japan; 3 Laboratory of Molecular Life Science, Institute of Biomedical Research and Innovation, Kobe 650-0047, Japan; 4 Department of Physiology, Keio University School of Medicine, Tokyo 160-8582, Japan

**Keywords:** Arf6, layer formation, membrane trafficking, migration, N-cadherin, receptor recycling

## Abstract

During neural development, endosomal trafficking controls cell shape and motility through the polarized transport of membrane proteins related to cell–cell and cell–extracellular matrix interactions. ADP ribosylation factor 6 (Arf6) is a critical small GTPase that regulates membrane trafficking between the plasma membrane and endosomes. We herein demonstrated that the knockdown of endogenous Arf6 in mouse cerebral cortices led to impaired neuronal migration in the intermediate zone and cytoplasmic retention of N-cadherin and syntaxin12 in migrating neurons. Rescue experiments with separation-of-function *Arf6* mutants identified Rab11 family-interacting protein 3 (FIP3)/Arfophilin-1, a dual effector for Arf6 and Rab11, as a downstream effector of Arf6 in migrating neurons. The knockdown of FIP3 led to impaired neuronal migration in the intermediate zone and cytoplasmic retention of N-cadherin in migrating neurons, similar to that of Arf6, which could be rescued by the coexpression of wild-type FIP3 but not *FIP3* mutants lacking the binding site for Arf6 or Rab11. These results suggest that Arf6 regulates cortical neuronal migration in the intermediate zone through the FIP3-dependent endosomal trafficking.

## Significance Statement

Endosomal trafficking is a critical mechanism that regulates cell motility and shape. During cortical layer formation, migrating neurons undergo dynamic changes in cell motility and shape, but regulatory mechanisms that control endosomal trafficking in migrating neurons have just begun to be elucidated. Here, we demonstrate that ADP ribosylation factor 6 (Arf6) small GTPase regulates neuronal migration in the intermediate zone through FIP3/Arfophilin-1-dependent endosomal transport of N-cadherin. The present findings indicate the importance of the Arf6-FIP3 pathway in the cortical layer formation and the existence of multiple small GTPases that regulate the surface N-cadherin expression in migrating neurons.

## Introduction

Neuronal migration is essential for the construction of brain structure and contributes to the evolutionary expansion of the number of neurons and complexity of neural circuits in the mammalian brain (Olson and Walsh, 2002; LoTurco and Bai, 2006; Rakic, 2007). Among various brain regions, the cerebral cortex has been extensively studied for neuronal migration due to its characteristic birthdate-dependent laminated structures and the feasibility of gene transfer by *in utero* electroporation (Inoue and Krumlauf, 2001; Saito and Nakatsuji, 2001; Tabata and Nakajima, 2001). Recent imaging analyses identified distinct migratory modes of radial migration during cortical development: multipolar migration, locomotion, and terminal translocation (Nadarajah et al., 2001; Tabata and Nakajima, 2003; Nishimura et al., 2010; Sekine et al., 2011; Ohshima, 2014).

Among these, multipolar migration is highlighted by its vulnerability, which causes neurodevelopmental disorders, including periventricular nodular heterotopia, subcortical band heterotopia, and double cortex syndrome (Gressens, 2000; Kato and Dobyns, 2003; Lu and Sheen, 2005; LoTurco and Bai, 2006; Cooper, 2014). During multipolar migration, neurons unsteadily move in the subventricular zone (SVZ) and intermediate zone (IZ) with their processes repeatedly extending and retracting, and establish cell polarity by forming an axon and reorienting intracellular organelles, such as the centrosome and Golgi apparatus (de Anda et al., 2010; Jossin, 2011; Sakakibara et al., 2014). In the upper IZ, multipolar neurons initiate contact with radial glial fibers, transform into a bipolar shape, and enter the locomotion mode (Tabata and Nakajima, 2003; Nishimura et al., 2010). Therefore, to complete multipolar-to-bipolar transition, multipolar cells may sense some directional cues through cell–cell and cell–extracellular matrix interactions. Consistent with this idea, recent evidence suggests that the surface expression of N-cadherin, a neural transmembrane cell adhesion molecule, on multipolar cells at an appropriate level and location is required for the multipolar-to-bipolar transition and regulated by endosomal trafficking mediated by Rap1 and Rab small GTPases (Kawauchi et al., 2010; Jossin and Cooper, 2011).

The ADP ribosylation factor (Arf) family is also a critical small GTPase for endosomal trafficking, and is grouped into three classes based on structural similarities: Arf1, Arf2, and Arf3 in class I; Arf4 and Arf5 in class II; and Arf6 in class III (D'Souza-Schorey and Chavrier, 2006; Gillingham and Munro, 2007; Donaldson and Jackson, 2011). Of these, Arf6 is present at the plasma membrane and a subpopulation of endosomes, where it regulates not only actin cytoskeleton remodeling but also endocytosis and/or the recycling of various receptors, including E-cadherin (Palacios et al., 2001, 2002), integrin (Powelka et al., 2004; Dunphy et al., 2006), transferrin receptor (D'Souza-Schorey et al., 1995), G-protein-coupled receptors (Claing et al., 2001; Houndolo et al., 2005; Macia et al., 2012), and major histocompatibility complex class I molecules (Klein et al., 2006).

Accumulating evidence implicates Arf6 as a critical regulator of cell shape and motility in various cell types. For example, the activation of Arf6 leads to the disassembly of adherens junctions through the internalization of E-cadherin, leading to changes in cell shape and motility, a process referred to as epithelial–mesenchymal transition, during wound healing and cancer invasion (Palacios et al., 2001, 2002; Luton et al., 2004). Arf6 also regulates the cell motility of MDA-MB231 breast cancer cells through the recycling of integrin β to the cell surface (Powelka et al., 2004), suggesting the importance of the Arf6-mediated polarized transport of cell adhesion molecules, such as cadherin and integrin, during cell migration and cancer invasion. Regarding the role of Arf6 in the developing cerebral cortex, Falace et al. (2014) provided the first evidence for the functional involvement of Arf6 in cortical neuronal migration. However, our understanding of how Arf6 regulates neuronal migration is still incomplete.

Here, we demonstrate that Arf6 regulates neuronal migration in the IZ through the interaction with Rab11 family-interacting protein 3 (FIP3), also known as Arfophilin-1 or Eferin, a dual effector of Arf6 and Rab11 for endosomal trafficking during cytokinesis (Fielding et al., 2005; Horgan and McCaffrey, 2009). We further demonstrate the functional involvement of the Arf6-FIP3 pathway in the trafficking of N-cadherin in cortical neurons. These results underscore the role of Arf6-FIP3-dependent endosomal trafficking in cortical neuronal migration.

## Materials and Methods

### Animals

Pregnant ICR (Institute for Cancer Research) mice were purchased from Charles River Japan. All experimental procedures in this study were approved by the Animal Experimentation and Ethics Committee of Kitasato University School of Medicine.

### Plasmid construction

The wild-type and mutant cDNAs for Arf6 (Arf6^T44N^ and Arf6^Q67L^) were kind gifts from Dr. Kazuhisa Nakayama at Kyoto University. The cDNAs for FIP3 and Rab11A were amplified by a polymerase chain reaction (PCR) from the mouse embryonic day (E) 17 brain cDNA library using the following primers supplemented with the EcoRI restriction site (underlined): 5′-GAA TTC ATG GAG CTG TGC CAG CCG ACC TCC C-3′ and 5′-CTA CTT GAC CTC TAG GAT GGA TGG GTT GGT C-3′ for FIP3; 5′-GAA TTC ATG GGC ACC CGC GAC GAG TA-3′ and 5′-TTA GAT GTT CTG ACA GCA CTG CAC CTT TGG-3′ for Rab11A. The PCR fragments were subcloned into the pGEM-T easy vector (Promega). FIP4 cDNA was purchased from DNAFORM (clone ID: B530005J18). The shRNA-resistant *Arf6* mutants (*Arf6^WT^*, *Arf6^iSW^*, *Arf6^W168L/L169V^*, and *Arf6^N48I^*), and *FIP3* mutants (*FIP3^WT^*, *FIP3^ΔABD^*, and *FIP3^ΔRBD^*) were made using the PrimeSTAR mutagenesis basal kit (Takara Bio) according to the manufacturer’s protocols. The resultant cDNAs were digested with EcoRI, and subcloned into the pCAGGS expression vector with the FLAG-tag sequence (Niwa et al., 1991; Sakagami et al., 2005) or pNeuroD-IRES-GFP vector, which had been provided by Dr. Franck Polleux at Columbia University via Addgene (plasmid #61403; Guerrier et al., 2009). Expression vectors carrying membrane-targeted and nucleus-targeted EGFP (EGFP-Fyn and EGFP-NLS) were constructed by subcloning the cDNAs for EGFP, which had been supplemented at C termini with a membrane-targeted signal from Fyn (MGCVQCKDKEATKLTEF) or nuclear localization signal (PKKKRKVEDA), respectively, into pCAGGS-loxP-polyA-loxP (Floxp; Shitamukai et al., 2011). pCAGGS-Cre-recombinase was a kind gift from Dr. Fumio Matsuzaki at the RIKEN Center for Developmental Biology. To construct shRNA vectors for Arf6 and FIP3, oligonucleotides targeting the coding sequence [5′-GCA CCG CAT TAT CAA TGA CCG-3′ for Arf6 KD, 5′-TCA CCT CAG GAG CAA GTA CCT-3′ for Arf6 KD scramble (Scr; Control), 5′-AAG CAG CTA GAA CAT CTA C-3′ for FIP3 KD#1, 5′-AAC CGT AAC CTG AAG GAG C-3′ for FIP3 KD#2, 5′-AAG GCT AAG GTC AAC CAC G-3′ for FIP3 KD#2 Scr (Control)] and their respective complementary sequences were aligned in tandem with a hairpin loop sequence (5′-TTC AAG AGA-3′), and inserted into the pmU6pro vector containing the mouse U6 small nuclear RNA promoter (Yu et al., 2002; Kawauchi et al., 2006). The target sequences were designed using the GenScript siRNA target finder (GenScript USA). All constructs using *in utero* electroporation were purified with PureLink HiPure Plasmid Filter Purification Kits (Life Technologies).

Regarding bacterial expression vectors for N-cadherin, the N-terminal extracellular region (amino acids 160–723) of mouse N-cadherin was amplified by PCR and subcloned into pGEX4T-2 (GE Healthcare) or pMAL-SXN, a modified pMAL-2c (New England Biolabs; Fukaya et al., 2011).

### *In situ* hybridization

The riboprobes for *in situ* hybridization against *Arf6*, *FIP3*, and *FIP4* were synthesized using the Digoxigenin RNA labeling Mix (Roche), and T7 and SP6 RNA polymerases (Promega). *In situ* hybridization was performed as described previously (Hara et al., 2013).

### Cell culture and transfection

To examine the efficiency of shRNAs, cultured cortical neurons were prepared as described previously (Beaudoin et al., 2012) and transfected before plating using Amaxa Nucleofector 2D (Lonza Group) according to the manufacturer’s protocol. Three days after plating, neurons were subjected to an immunoblot analysis to assess shRNA efficiency and internalization/recycling assay for N-cadherin.

### Antibodies

To produce an anti-Arf6 antibody, a 10 aa peptide (LTWLTSNYKS) corresponding to amino acids 166–175 of mouse Arf6 was conjugated to the Keyhole limpet hemocyanin (KLH) and used as an antigen for immunization and affinity purification. Regarding an anti-N-cadherin antibody, the N-terminal extracellular region (amino acids 160–723) of mouse N-cadherin was expressed as fusion proteins of glutathione S-transferase (GST) and maltose-binding protein (MBP) in *Escherichia coli* BL21 (DE3; Stratagene) in the presence of 0.3 mm isopropyl-β-D-thiogalactopyranoside at 25ºC overnight and purified with glutathione-Sepharose 4B (GE Healthcare) and amylose-resin (New England Biolabs), respectively. Rabbits and guinea pigs were subcutaneously immunized with the KLH-conjugated peptide or GST-N-cadherin fusion protein five times at 2 week intervals. Sera were affinity-purified using cyanogen bromide-activated Sepharose 4B (GE Healthcare) conjugated with the Arf6 peptide or MBP-N-cadherin fusion protein. The specificities of antibodies were characterized by an immunoblot analysis.

### Immunoblot analysis

Lysates (10 μg) were subjected to an immunoblot analysis with the following primary antibodies at a final concentration of 0.5 μg/ml: guinea pig anti-Arf6 IgG, guinea pig anti-FIP3 IgG (Yazaki et al., 2014), mouse anti-N-cadherin IgG (32/N-cadherin, BD Transduction Laboratories), and mouse anti-α-tubulin IgG (DM1A, Sigma-Aldrich). Immunoreactive bands were detected using ECL Plus kit (Thermo Fisher Scientific) and LAS-4000 miniluminescent image analyzer (GE Healthcare). The quantification of intensities for immunoreactive bands was performed using ImageJ software (National Institutes of Health).

### *In utero* electroporation

*In utero* electroporation was performed as described previously (Kawauchi et al., 2010; Hara et al., 2013). Plasmids were mixed under the following conditions in phosphate-buffered saline (PBS): overexpression experiments, 3 μg/μl of the plasmid; knockdown experiments, 1 μg/μl of the shRNA plasmid; rescue experiments, 1 μg/μl of the shRNA plasmid and 0.1 μg/μl of pCAGGD-Arf6-FLAG or 0.5 μg/μl of pCAGGS-FLAG-FIP3 in combination with 0.5 μg/μl of pCAGGS-mCherry or EGFP. Electroporation (35 V, 450 ms, three pulses) was performed for mouse embryos at E14.5 through the uterus with forceps-type electrodes using a square electroporator (CUY-21 Edit, Bex). The embryos were fixed at E15.5, E16.5, E17.5, or postnatal day (P) 0. To label proliferating cells, pregnant mice at E15.5 were intraperitoneally administrated 10 mg/ml of 5-bromo-2-deoxyuridine (BrdU, Roche) dissolved in 0.9% saline solution (140 mg/kg body weight) three times at 20 minute intervals before being killed. In time-lapse imaging, a mixture of the pCAGGS-Floxp plasmids encoding EGFP, EGFP-Fyn, and EGFP-NLS were electroporated together with shRNA and pCAGGS-Cre-recombinase.

### Immunohistology

Immunohistochemistry and BrdU staining were performed as described previously (Hara et al., 2013). The following primary antibodies were used at a final concentration of 1 μg/ml: guinea pig anti-Arf6 IgG, goat anti-MAP2 IgG (MAP2-Go-Af, Frontier Institute), goat anti-BLBP IgG (Yamada et al., 2000), rabbit anti-EEA1 IgG (Fukaya et al., 2014), rabbit anti-syntaxin12 (STX12) IgG (Hara et al., 2013), goat anti-LAMP2 polyclonal IgG (sc-8100, Santa Cruz Biotechnology), guinea pig or rabbit anti-mCherry IgG (Hara et al., 2013), rabbit anti-EGFP IgG (Sakagami et al., 2005), mouse anti-polysialated-neural cell adhesion molecule (anti-PSA-NCAM) IgM (Seki, 2002), rabbit anti-Rab11 IgG (71-5300, Life Technologies), guinea pig anti-FIP3 IgG (Yazaki et al., 2014), and guinea pig anti-N-cadherin IgG. The immunoreaction was visualized using species-specific secondary antibodies conjugated with Alexa488, Alexa594, or Alexa647 (Invitrogen). Nuclei were counterstained with 4′,6-diamidino-2-phenylindole (Roche). Immunoreactions were examined with a confocal laser microscope (LSM 710, Carl Zeiss).

### Quantitative analysis

The dorsolateral region of the cerebral cortex was analyzed for all electroporation experiments. One representative brain section was selected from each sample, and EGFP-positive or mCherry-positive cells in each cortical layer, which was identified based on cell density visualized by nuclear staining with DAPI, were counted in a blind manner using ImageJ software (National Institutes of Health). Data were collected from ≥3 animals for each condition as indicated in graphs. The fraction of EGFP-positive or mCherry-positive cells in each cortical layer of the brain transfected with the indicated plasmids was compared with that in the equivalent layer under the control conditions. Statistical analyses were performed using the paired *t* test for direct comparison, or one-way ANOVA with *post hoc* Tukey–Kramer’s test for multiple comparisons.

### Time-lapse imaging

Time-lapse imaging was performed as described previously (Tabata and Nakajima, 2003). Embryos were electroporated at E14.5 and collected at E17.5. Brains were embedded with 3% low melting temperature agarose gel and sliced into 200-μm-thick sections with a microslicer (VT1000S, Leica). The slices were placed on an insert membrane (Millipore) and maintained in glass-bottom dishes (Iwaki) filled with culture medium. Recording was carried out using a confocal laser microscope (LSM 710, Carl Zeiss) and stage top incubator (40% O_2_, 5% CO_2_; ZILCS-H2; Tokai Hit). Images were collected every 15 min for 20 h and analyzed using ImageJ software (National Institutes of Health).

### Internalization and recycling assays

Internalization and recycling assays were performed as described previously (Tai et al., 2007). Three days after transfection and plating, cultured cortical neurons were treated with 100 μg/ml leupeptin at 37°C for 1 h. Cell-surface proteins were labeled with 0.3 mg/ml of Sulfo-NHS-SS-Biotin (Thermo Fisher Scientific) in PBS containing Mg^2+^ and Ca^2+^ (PBS-MC) on ice for 12 min. Excess biotin was quenched by Tris buffer (25 mm TrisHCl, pH 8.0, 133 mm NaCl, 10 mm KCl). Neurons were then rinsed twice with maintenance medium (Neurobasal medium, 1× B27 supplement, 2 mm L-glutamine, penicillin/streptomycin) and incubated at 37°C for various periods as indicated in Figure 4D. At the end of each time point, neurons were chilled to 4°C by washing with ice-cold PBS-MC, and biotins exposed on the cell surface were removed by rinsing twice for 15 min with ice-cold 50 mm glutathione solution (50 mm reduced glutathione, 75 mm NaCl, 10 mm EDTA, 1% BSA, pH 8.0). Neurons were then solubilized with lysis buffer (0.1 m phosphate buffer, pH 7.5, 150 mm NaCl, 1% Triton X-100, 0.1% SDS) containing a mixture of protease inhibitors (Sigma-Aldrich) and phenylmethylsulfonyl fluoride. Internalized biotinylated membrane proteins were precipitated with NeutrAvidin resin (Thermo Fisher Scientific) overnight at 4°C and subjected to an immunoblot analysis with anti-N-cadherin IgG (BD Transduction Laboratories).

In the recycling assay, neurons were biotinylated and incubated for 90 min at 37°C, and biotinylated proteins remaining on the cell surface were removed by rinsing with glutathione solution. Neurons were then incubated for the various periods indicated in Figure 4E. At the end of each time point, neurons were treated with glutathione solution and lysed. Internalized biotinylated membrane proteins were precipitated with NeutrAvidin resin (Thermo Fisher Scientific) and subjected to an immunoblot analysis. The significance of differences was determined by the Mann-Whitney *U* test.

## Results

### Arf6 is required for cortical layer formation

**Figure 1.**
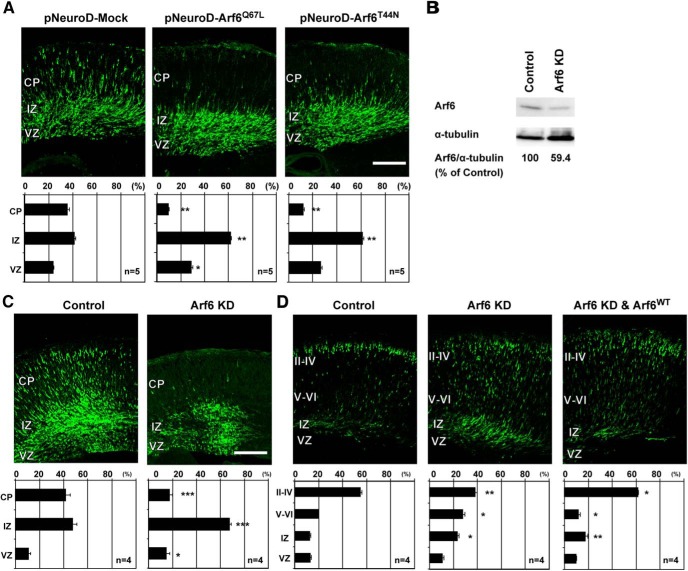
**Arf6 is required for cortical neuronal migration. *A***, Representative micrographs of E17.5 cerebral cortices electroporated with the pNeuroD plasmids carrying Mock, Arf6^Q67L^, or Arf6^T44N^ in combination with pCAGGS-EGFP at E14.5 (*n* = 5 embryos). ***B***, Immunoblot analysis of cultured cortical neurons transfected with Arf6 shRNA (Arf6 KD) or its scramble shRNA (Control) for 3 d. The numbers indicate the percentage of endogenous Arf6 expression level relative to α-tubulin. ***C***, Representative micrographs of E17.5 cerebral cortices electroporated with the pmU6pro plasmids carrying scramble sequence (Control) or Arf6 shRNA (Arf6 KD) in combination with pCAGGS-EGFP at E14.5 (*n* = 4 embryos). ***D***, Representative micrographs of P0 cerebral cortices electroporated with Control, Arf6 KD, or Arf6 KD and Arf6^WT^ plasmids in combination with pCAGGS-EGFP at E14.5 (*n* = 4 embryos). Bottom graphs in ***A***, ***C***, and ***D*** show the quantification of the distribution of EGFP-positive cells in cortical layers. Data were presented as mean ± SEM and statistically analyzed using one-way ANOVA followed by Tukey–Kramer’s tests in ***A*** and ***D*** (*P < 0.05, **P < 0.01), and by unpaired *t* test in ***C*** (*P < 0.05, **P < 0.005, ***P < 0.0005). *n* in the graph indicates the number of embryos examined. Scale bars: ***A***, ***C***, ***D***, 200 μm.

In a previous study, overexpression of constitutively active Arf6 mutant *Arf6^Q67L^* in cortical progenitor cells under a ubiquitous promoter was shown to disturb cortical layer formation (Falace et al., 2014). However, it remains unclear whether this effect may be attributed to the overactivation of Arf6 in neurons, glial cells, or both. In order to understand the role of Arf6 in neuronal migration in more detail, we first examined the effects of overexpression of *Arf6* mutants specifically in postmitotic neurons under the NeuroD promoter (Guerrier et al., 2009). At E17.5, three days after *in utero* electroporation, significantly more neurons overexpressing either *Arf6^Q67L^* or GDP-locked inactive mutant *Arf6^T44N^* were retained in the IZ than control neurons (Fig. 1A; Control, 41.2 ± 1.9%; *Arf6^T44N^*, 61.3 ± 1.5%, *P* < 0.01; *Arf6^Q67L^*, 61.6 ± 2.3%, *P* < 0.01), suggesting the importance of the proper functioning of the GDP/GTP cycling of Arf6 in migrating neurons for cortical layer formation.

Next, we examined the effect of the knockdown of endogenous Arf6 on cortical layer formation using shRNA. In the first step to determine the efficiency of shRNA for Arf6, cultured cortical neurons from E14.5 cerebral cortices were transfected with shRNA and harvested 3 d after transfection. The immunoblot analysis revealed that the expression of Arf6 shRNA significantly decreased endogenous Arf6 in cultured cortical neurons by ∼40% compared with that of control scramble shRNA (Fig. 1B). Having established the efficiency of Arf6 shRNA, we introduced Arf6 shRNA together with EGFP into ventricular progenitor cells in the dorsal pallium at E14.5 by *in utero* electroporation and analyzed the distribution of EGFP-positive cells in the cerebral cortex 3 and 5 d later. At E17.5, 41.7 ± 3.7% of control neurons were migrating in the cortical plate (CP) with a bipolar shape, whereas 62.5 ± 0.7% of Arf6-knockdown neurons were still positioned in the IZ with only 21.2 ± 1.8% in the CP (Fig. 1C), suggesting that the knockdown of Arf6 delayed the exit of migrating neurons from the IZ to the CP. At P0, the knockdown of Arf6 led to a pronounced migration defect with a significantly smaller proportion of neurons reaching the superficial layers II–IV than control neurons (Fig. 1D; Control, 54.9 ± 1.2%; Arf6 knockdown, 38.3 ± 1.6%, *P* < 0.01, *n* = 4 embryos). In a rescue experiment, the coexpression of shRNA-resistant wild-type Arf6 (Arf6^WT^), in which four target nucleotides were mutated without amino acid substitutions, with Arf6 shRNA restored the proportion of cells that reached the superficial layers at P0 to an indistinguishable level from the control (Fig. 1D), suggesting the target specificity of Arf6 shRNA.

**Figure 2.**
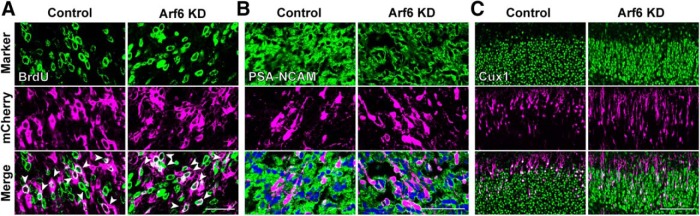
**Effects of the knockdown of Arf6 on cell proliferation and neuronal differentiation. *A***, Representative micrographs showing the distribution of BrdU-positive cells in the VZ. Embryos were electroporated with the indicated shRNAs and EGFP at E14.5 and killed at E15.5 1 h after the intraperitoneal administration of BrdU to pregnant mice. Arrowheads show BrdU-incorporated mCherry-positive cells in the VZ. Note no apparent differences in the cell number or position of BrdU-positive transfected cells between Control and Arf6 knockdown (Arf6 KD). ***B***, Representative micrographs showing the expression of PSA-NCAM in control and Arf6-KD neurons in the IZ at E17.5. ***C***, Representative micrographs showing that the expression of Cux1 in control and Arf6-KD neurons in the superficial cortical layers at P0. Scale bars: ***A***, 25 μm; ***B***, 50 μm; ***C***, 100 μm.

Since Arf6 is known to regulate cytokinesis during mitosis (Schweitzer and D'Souza-Schorey, 2002, 2005), we examined the possibility that the knockdown of Arf6 could affect the cell proliferation in the ventricular zone (VZ)/SVZ. Pregnant mice, which had been subjected to *in utero* electroporation with pCAGGS-EGFP at E14.5, were intraperitoneally administrated BrdU 3 times at E15.5 and embryos were fixed 1 h later. BrdU immunostaining failed to detect any apparent differences in the number or position of BrdU-incorporated transfected cells between Arf6-knockdown and control cells in the VZ (Fig. 2A; Control, 36.9 ± 0.1%; Arf6 knockdown, 33.6 ± 0.1%, *P* = 0.61, *n* = 3 embryos). Further immunostaining of cerebral cortices, which had been electroporated with control or Arf6 shRNA, with neuronal differentiation markers failed to detect any apparent differences in the expression of PSA-NCAM, a marker for immature neurons, in the IZ at E17.5 (Fig. 2B; Control, 93.6 ± 0.01%; Arf6 knockdown, 89.5 ± 0.1%, *P* = 0.21, *n* = 3 embryos), or Cux1, a marker for cortical superficial layers, in the CP at P0 (Fig. 2C; Control, 90.7 ± 0.04%; Arf6 knockdown, 90.5 ± 0.1%, *P* = 0.95, *n* = 3 embryos) between control and Arf6-knockdown cells, excluding the possibility that the knockdown of Arf6 affected neuronal differentiation. Together, these results suggest that not only the proper GDP/GTP cycling but also the endogenous expression of Arf6 is required for the cortical layer formation.

### Arf6 regulates neuronal migration in the IZ

To determine how Arf6 regulates the properties of neuronal migration, we performed the time-lapse imaging analysis of neuronal migration using acute cortical slices at E17.5, 3 d after *in utero* electroporation. To monitor the cell behavior at the single-cell level, shRNA-transfected neurons were sparsely labeled by a mixture of cytoplasm-targeted, nucleus-targeted, and membrane-targeted EGFP using a Cre-loxP clonal expression system (Shitamukai et al., 2011).

**Figure 3.**
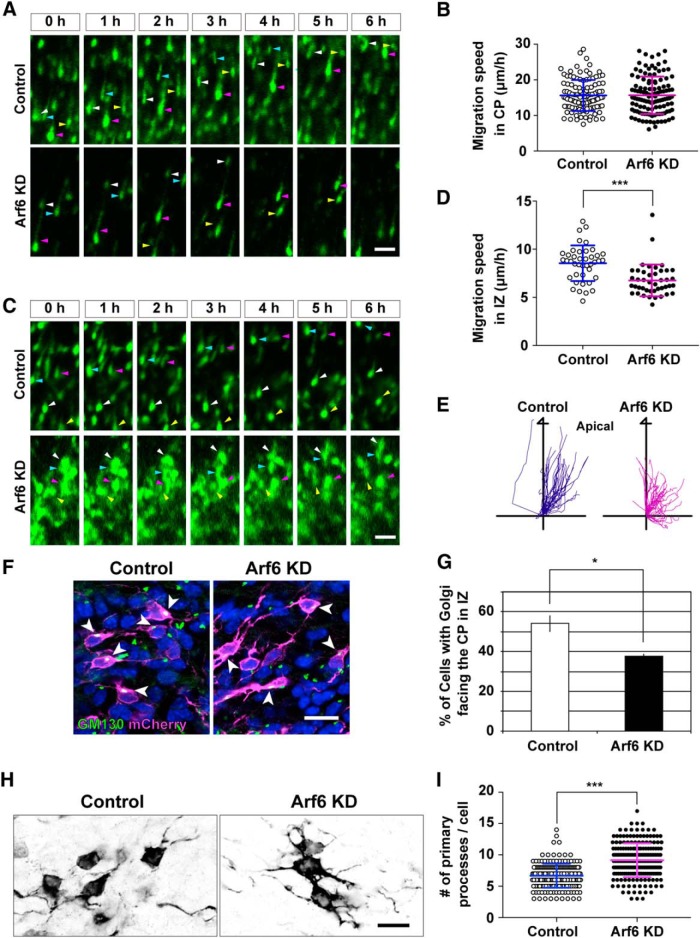
**Arf6 is important for neuronal migration in the IZ. *A***, ***C***, Representative time-lapse images of transfected migrating neurons in the CP and IZ. Embryos were electroporated with the indicated shRNA in combination with pCAGGS-Cre recombinase and pCAGGS-Floxp carrying EGFP, EGFP-NLS, and EGFP-Fyn at E14.5, and brains were subjected to slice culture and time-lapse observations at E17.5. Arrowheads indicate transfected neurons. ***B***, ***D***, Quantification of the migration speed of transfected neurons in the CP (***B***, *n* = 115 cells from 3 embryos) and IZ [***D***, Control, *n* = 41 cells from 3 embryos; Arf6 knockdown (Arf6 KD), *n* = 43 cells from 3 embryos]. ***E***, Tracking analysis of migration of transfected neurons in the IZ. The graphs show the trajectory of individual migrating neurons (Control, 41 cells; Arf6 KD, 43 cells from 3 embryos) for 10–13 h. ***F***, The orientation of the Golgi apparatus in migrating neurons in the IZ. Arrowheads indicate the Golgi apparatus labeled by GM130 in transfected neurons in the IZ. ***G***, Quantification of the proportion of cells in the IZ with the Golgi apparatus facing the CP. Note the disorientation of Golgi apparatus in Arf6-KD neurons (*n* = 3 embryos). ***H***, Representative micrographs showing the morphology of transfected neurons in the IZ at E16.5. Brains were electroporated with the indicated shRNA plasmids in combination with pCAGGS-EGFP at E14.5 and immunostained with an anti-EGFP antibody. ***I***, Quantification of the number of primary processes. Note the increase in primary processes in migrating neurons transfected with Arf6-KD plasmid (Control, *n* = 195 cells from 3 embryos; Arf6 KD, *n* = 208 cells from 3 embryos). Data were presented as mean ± SD, and statistically analyzed by unpaired *t* test (*P < 0.05, ***P < 0.0005). Scale bars: ***A***, ***C***, 50 μm; ***F***, ***H***, 20 μm.

In the CP, control and Arf6-knockdown neurons radially migrated toward the pial surface with a typical bipolar shape, as shown in Figure 3A. The morphology and migration speed of radially migrating neurons were indistinguishable between control and Arf6-knockdown neurons (Fig. 3A,*B*; Control, 15.6 ± 0.4 μm/h; Arf6 KD, 15.7 ± 0.5 μm/h, *P =* 0.9203). By contrast, in the IZ, the migration speed toward the CP was significantly slower in Arf6-knockdown neurons than in the control neurons (Fig. 3C,*D*; Control, 8.5 ± 0.3 μm/h; Arf6 KD, 6.8 ± 0.3 μm/h, *P* < 0.0005). Furthermore, a single-cell tracking analysis revealed that directional migration of Arf6-knockdown neurons toward the CP in the IZ was disorganized compared with that of the control neurons (Fig. 3E). These results suggest that Arf6 regulates neuronal migration in the IZ but not in the CP during cortical layer formation.

Since the position of intracellular organelles, such as the centrosome and Golgi apparatus is reoriented during the multipolar-to-bipolar transition in the IZ (Jossin, 2011; Sakakibara et al., 2014), we investigated the position of the Golgi apparatus in multipolar neurons in the IZ at E16.5 by immunostaining with an anti-GM130 antibody, a marker for the *cis*-Golgi. The knockdown of Arf6 significantly decreased the proportion of multipolar neurons with the Golgi apparatus oriented toward the CP compared with the control scramble shRNA (Fig. 3F,*G*; Control, 54.1 ± 4.2%; Arf6 knockdown, 37.7 ± 1.4%, *P <* 0.05). Furthermore, the number of primary processes increased in Arf6-knockdown neurons (Fig. 3H,*I*; Control, 6.7 ± 0.2; Arf6 knockdown, 9.0 ± 0.6, *P <* 0.0005). These results suggest that Arf6 regulates the establishment of cell polarity in migrating neurons during the multipolar-to-bipolar transition in the IZ.

### Arf6 regulates the transport of recycling endosomes in migrating neurons

**Figure 4.**
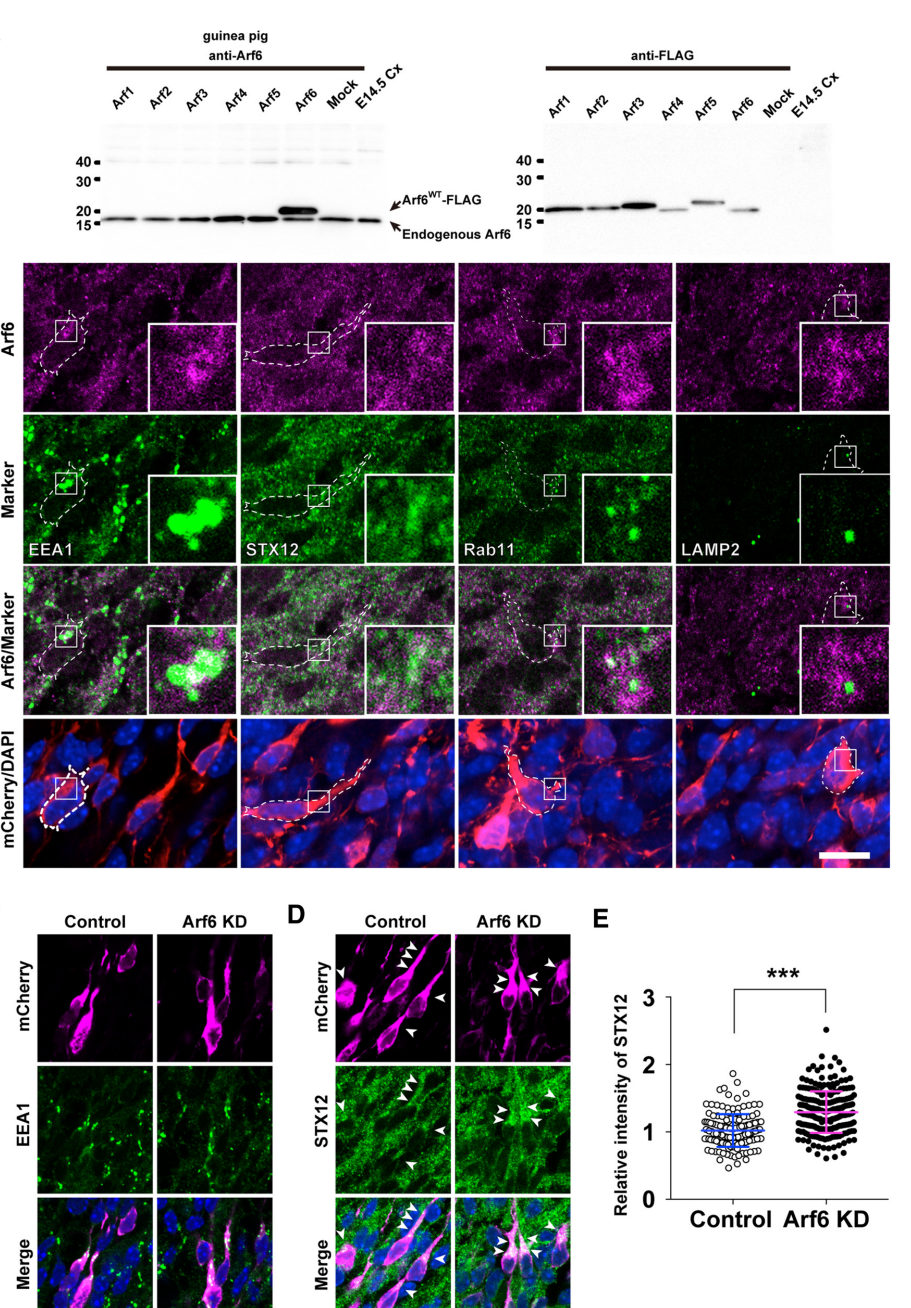
**Arf6 localizes at endosomes and regulates the distribution of syntaxin12-positive endosomes in migrating neurons. *A***, Immunoblot analysis showing the specificity of rabbit anti-Arf6 antibody. The lysates of the mouse cerebral cortex (Cx) at E14.5 (10 μg) and HeLa cells transfected with the indicated FLAG-tagged Arf plasmids were immunoblotted with antibodies against Arf6 or FLAG. The positions and sizes of the molecular weight markers are indicated on the left. ***B***, Representative micrographs showing endosomal localization of Arf6 in migrating neurons in the IZ at E17.5. Coronal sections of the E17.5 cerebral cortex, which had been electroporated with pCAGGS-mCherry at E14.5, were subjected to immunofluorescent staining with antibodies against Arf6 (magenta), the indicated markers (green), and mCherry (red). Nuclei were counterstained with DAPI (blue). Insets show the high-magnification views of boxed areas. ***C***, ***D***, Representative micrographs showing the subcellular localization of EEA1-positive (***C***) and syntaxin12 (STX12)-positive (***D***) endosomes in migrating neurons transfected with Control or Arf6 knockdown (Arf6 KD) plasmid in the IZ at E17.5. Arrowheads in ***D*** indicate STX12-positive puncta in transfected neurons. ***E***, Quantification of the relative immunofluorescence intensity for STX12 in the perinuclear region. The relative immunofluorescence intensity was calculated by normalizing the immunofluorescence intensity for perinuclear STX12 in transfected neurons to that in the surrounding untransfected neurons. Note the significant increase in perinuclear STX12 in Arf6-KD neurons (Control, *n* = 155 cells; Arf6 KD, *n* = 239 cells). Data collected from three embryos in each condition were presented as mean ± SD and statistically analyzed with unpaired *t* test (*** *P* < 0.0001). Scale bars: ***B–D***, 10 μm.

To obtain a mechanistic insight into the role of Arf6 in neuronal migration, we first examined the subcellular localization of Arf6 in migrating neurons using a novel anti-Arf6 antibody raised against the C-terminal 10 aa sequence, which is divergent among the Arf family. In the immunoblot analysis, the anti-Arf6 antibody specifically detected a 17-kDa immunoreactive band corresponding to endogenous human Arf6 in HeLa cell lysates and an additional band in the lysates of HeLa cells expressing FLAG-tagged mouse Arf6, but not other Arf isoforms (Fig. 4A). Having confirmed the specificity of the antibody, we performed double immunofluorescent staining of migrating neurons at E17.5, which had been transfected with mCherry at E14.5, with antibodies against Arf6 and various endosomal markers: EEA1 for early endosomes, STX12 and Rab11 for recycling endosomes, and LAMP2 for late endosomes and lysosomes. The immunofluorescene for Arf6 appeared as numerous cytoplasmic fine puncta in migrating neurons and partially colocalized with EEA1, STX12, and Rab11, but not with LAMP2 (Fig. 4B). These results suggest the occurrence of Arf6 at subpopulations of early and recycling endosomes in migrating neurons in the IZ.

This subcellular localization of Arf6 prompted us to examine the effect of the knockdown of Arf6 on the endosomal system in migrating neurons by immunostaining with endosomal markers. Arf6-knockdown and control neurons in the IZ at E17.5 exhibited indistinguishable intracellular distribution of EEA1-positive early endosomes (Fig. 4C). On the other hand, Arf6-knockdown neurons, compared with control neurons, had more STX12-positive endosomes accumulated in the perinuclear region (Fig. 4D). A quantitative analysis revealed that the ratio of the fluorescence intensity for perinuclear STX12 in transfected cells to that in the surrounding untransfected cells in the same sections was significantly higher in Arf6-knockdown neurons than in control neurons (Fig. 4E; Control, 1.0 ± 0.2; Arf6 KD, 1.3 ± 0.2, *P* < 0.0001). These results suggest that the knockdown of Arf6 disturbs the transport of a subpopulation of recycling endosomes in migrating neurons in the IZ.

### Arf6 regulates the recycling of N-cadherin in cortical neurons

**Figure 5.**
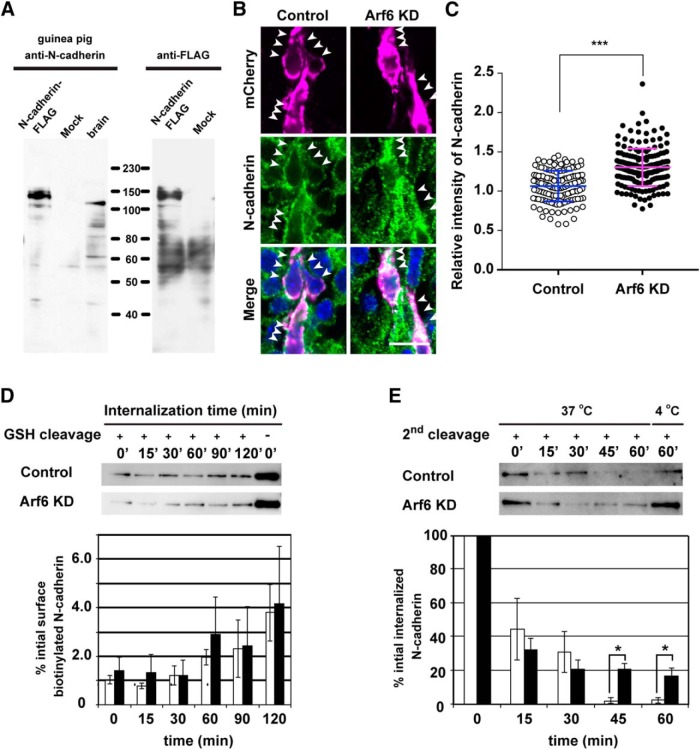
**Arf6 regulates endosomal trafficking of N-cadherin in cortical neurons. *A***, Immunoblot analysis showing the specificity of guinea pig anti-N-cadherin antibody. The lysates of the adult mouse brain (10 µg) and HeLa cells transfected with or without C-terminally FLAG-tagged N-cadherin were immunoblotted with antibodies against N-cadherin or FLAG. ***B***, Representative micrographs showing the subcellular localization of N-cadherin in migrating neurons transfected with Control or Arf6 knockdown (Arf6 KD)in the IZ at E17.5. Arrowheads indicate the localization of N-cadherin in transfected neurons. ***C***, Quantification of the relative immunofluorescence intensity for cytoplasmic N-cadherin. The relative intensity was calculated by normalizing the immunofluorescence intensity for cytoplasmic N-cadherin in transfected neurons to that in the surrounding untransfected neurons (Control, *n* = 137 cells from 3 embryos; Arf6 KD, 174 cells from 3 embryos). Note the significant increase in cytoplasmic N-cadherin in Arf6-KD neurons. ***D***, Internalization assay for N-cadherin in cultured cortical neurons. A bottom graph shows the quantification of biotinylated N-cadherin internalized in neurons transfected with Control (open bars) or Arf6 KD(black bars; Control, *n* = 5 independent experiments; Arf6 KD, *n* = 5). ***E***, Recycling assay for N-cadherin in cultured cortical neurons. A bottom graph shows the quantification of biotinylated N-cadherin retained inside neurons transfected with Control (open bars) or Arf6 KD (black bars; Control, *n* = 5 independent experiments; Arf6 KD, *n* = 6). Data were presented as mean ± SD in ***C*** and mean ± SEM in ***D*** and ***E***, and statistically analyzed by unpaired *t* test in ***C*** (***P < 0.0001), and Mann-Whitney *U* test in ***D*** and ***E*** (*P < 0.05). GSH, Glutathione. Scale bar: ***B***, 20 μm.

Recent evidence highlighted the functional importance of endosomal trafficking of cell adhesion molecules, such as N-cadherin and integrin β, in neuronal migration (Jossin, 2011; Kawauchi, 2012; Sekine et al., 2014). Particularly, Rap1 and Rab small GTPases were shown to regulate cortical neuronal migration through the endosomal trafficking of N-cadherin (Kawauchi et al., 2010; Jossin and Cooper, 2011). In addition, Arf6 is implicated in cell shape and motility during cancer invasion and wound healing through the regulation of surface E-cadherin expression (Palacios et al., 2001, 2002). Therefore, we hypothesized that Arf6 may be functionally linked to the endosomal trafficking of N-cadherin in migrating neurons. To test this hypothesis, we examined the subcellular localization of N-cadherin in migrating neurons by immunostaining with an anti-N-cadherin antibody raised against its extracellular domain (Fig. 5A). In the control neurons migrating in the IZ, the immunofluorescence for N-cadherin was largely distributed along the plasma membrane (Fig. 5B). By contrast, the knockdown of Arf6 resulted in the accumulation of the immunofluorescence for N-cadherin in the cell bodies of transfected neurons (Fig. 5B). A quantitative analysis confirmed that the immunofluorescence for cytoplasmic N-cadherin was significantly higher in Arf6-knockdown neurons than in control neurons (Fig. 5C; Control, 1.1 ± 0.2; Arf6 KD, 1.3 ± 0.2, *P* < 0.0001).

To further verify the involvement of Arf6 in the trafficking of N-cadherin in cortical neurons, we investigated the effects of the knockdown of Arf6 on the time course of the internalization and recycling of N-cadherin in cultured cortical neurons from E14.5 mouse embryos. In the internalization assay, cultured cortical neurons transfected with control or Arf6 shRNAs were subjected to surface labeling with cleavable biotin, followed by internalization in the presence of leupeptin to inhibit lysosomal proteolysis. At various time points, the remaining surface biotin was removed and internalized biotinylated proteins were precipitated by avidin-conjugated resin. The immunoblot analysis with an anti-N-cadherin antibody revealed that the time course and ratio of N-cadherin internalization were indistinguishable between control and Arf6-knockdown neurons (Fig. 5D). In the recycling assay, after biotinylated neurons were subjected to internalization at 37ºC for 90 min, the biotin recycled to the cell surface was removed at various time points, and the biotinylated proteins retained in the cell body were precipitated by avidin-conjugated resin and subjected to an immunoblot analysis. In control neurons, internalized biotinylated N-cadherin was recycled back to the plasma membrane at 45 min, whereas ∼20% of internalized N-cadherin was still retained in the cell bodies of Arf6-knockdown neurons, even at 60 min (Fig. 5E; 45 min: Control, 2.0 ± 1.7%; Arf6 knockdown, 20.6 ± 3.4%, *P =* 0.011; 60 min: Control, 2.3 ± 1.7%; Arf6 knockdown, 16.5 ± 4.8%, *P =* 0.028). These results suggest that Arf6 regulates the recycling of N-cadherin in cortical neurons.

### FIP3 is a candidate for Arf6 downstream effector in neuronal migration.

**Figure 6.**
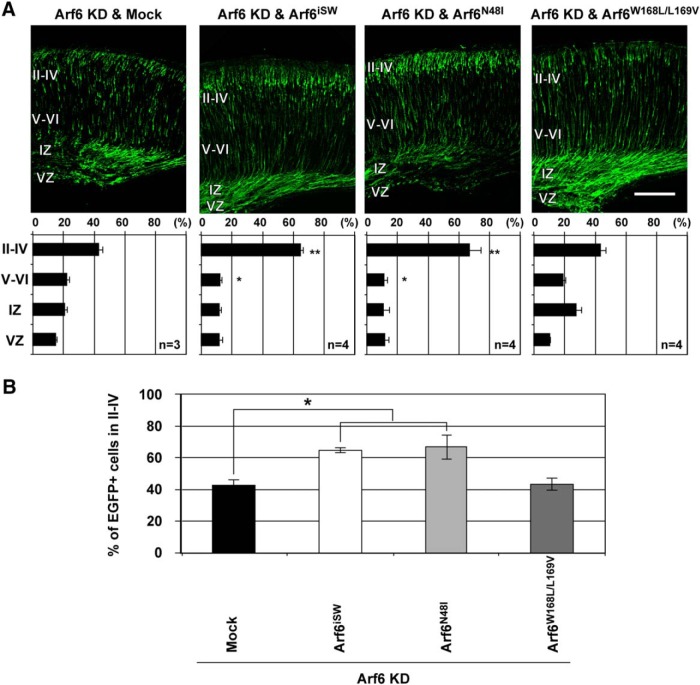
**Arf6 regulates neuronal migration through the interaction with class II FIPs. *A***, Representative micrographs showing rescue experiments of impaired neuronal migration caused by the knockdown of Arf6 with separation-of-function *Arf6* mutants. Brains were electroporated with the indicated pCAGGS plasmids carrying Mock, Arf6^iSW^, Arf6^N48I^, Arf6^W168L/L169V^ in combination with Arf6 shRNA and pCAGGS-EGFP at E14.5, and fixed at P0. Bottom graphs show the quantification of the distribution of EGFP-positive cells in cortical layers. ***B***, Comparison of the percentage of EGFP-positive cells transfected with the indicated plasmids in the superficial layers II–IV at P0. Data were presented as mean ± SEM and statistically analyzed using one-way ANOVA followed by *post hoc* Tukey-Kramer’s test (*P < 0.05). *n* in the graph indicates the number of embryos examined. Scale bar, 200 μm.

To elucidate the Arf6 downstream pathways that regulate neuronal migration in more detail, we examined whether the impaired neuronal migration phenotype caused by Arf6 shRNA could be rescued by the coexpression of separation-of-function *Arf6* mutants that interfere with the interaction with specific downstream effectors: *Arf6^iSW^*, which blocks the interaction with JIP3/4 and vezatin by mutating *Asn-45*, *Thr-53*, *Val-57*, *Lys-58*, and *Asn-60* residues in the interswitch region (Montagnac et al., 2009; Sanda et al., 2010; Suzuki et al., 2010); *Arf6^N48I^*, which blocks the Arf6-dependent activation of phospholipase D by mutating *Asn-48* to *Ile* (Vitale et al., 2005); *Arf6^W168L/L169V^*, which blocks the interaction with class II FIPs by mutating *Trp-168* and *Leu-169* to *Leu* and *Val*, respectively (Schonteich et al., 2007). At P0, the cotransfection of either *Arf6^iSW^* or *Arf6^N48I^* with Arf6 shRNA significantly increased the proportion of EGFP-positive cells in the superficial cortical layer compared with Arf6 knockdown (Fig. 6A,*B*; Arf6 knockdown and Mock, 34.3 ± 5.0%; Arf6 knockdown and *Arf6^iSW^*, 66.7 ± 2.8%, *P* < 0.05; Arf6 knockdown and *Arf6^N48I^*, 68.8 ± 5.8%, *P* < 0.05). On the other hand, the cotransfection of *Arf6^W168L/L169V^* with Arf6 shRNA did not restore the delay in neuronal migration with only 38.4 ± 3.1% of EGFP-positive neurons in the superficial cortical layers (Fig. 6A,*B*). These results suggest that Arf6 may regulate neuronal migration through the interaction with class II FIPs.

**Figure 7.**
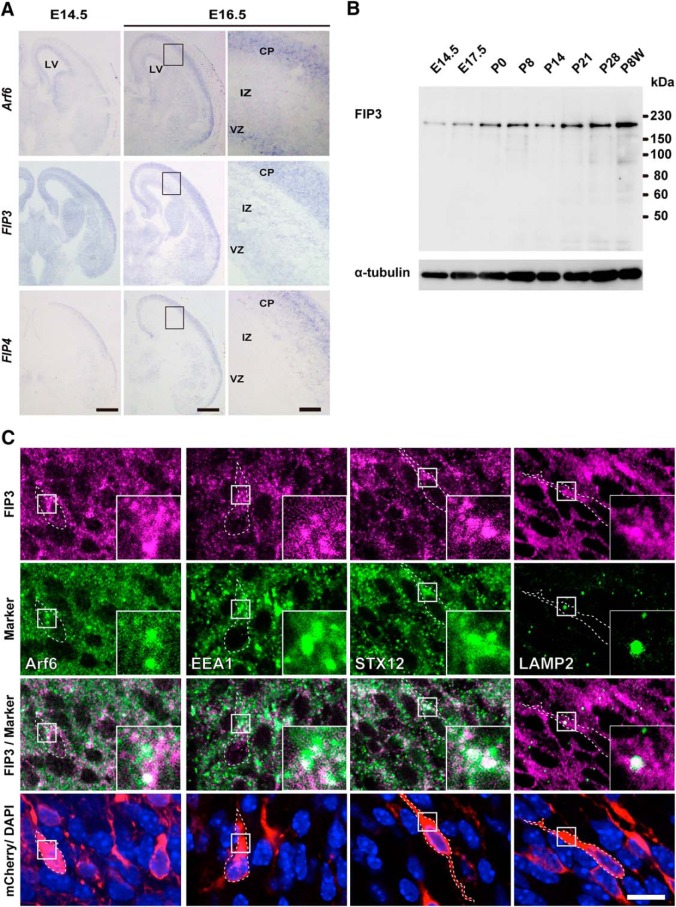
**Expression of FIP3 in the developing cerebral cortex. *A***, Representative micrographs showing the gene expression of *Arf6*, *FIP3*, and *FIP4* in the developing cerebral cortex. Coronal sections of mouse cerebral cortices at E14.5 and E16.5 were subjected to nonradioactive *in situ* hybridization analysis for *Arf6*, *FIP3*, and *FIP4*. Right, Magnified views of the boxed region in the middle panel. Note the widespread gene expression of *FIP3* and *Arf6* in the VZ, IZ, and CP in contrast to the preferential gene expression of *FIP4* in the CP. ***B***, Immunoblot analysis of developing cerebral cortices with anti-FIP3 and anti-α-tubulin antibodies. Note the gradual increase in FIP3 during cortical development. The positions and sizes of the molecular weight markers are indicated on the right. ***C,*** Representative micrographs showing the subcellular localization of FIP3 in migrating neurons in the IZ at E17.5. Coronal sections of the cerebral cortex, which had been electroporated with pCAGGS-mCherry at E14.5, were subjected to immunofluorescent staining with antibodies against FIP3 (magenta), the indicated markers (green), and mCherry (red). Nuclei were counterstained with DAPI (blue). Insets show the high-magnification views of boxed areas. Scale bars: ***A***, left, middle, 500 μm; ***A***, right, 10 μm; ***C***, 20 μm.

The class II FIP family includes two structurally related isoforms, FIP3 and FIP4, and is known to function as an effector of Arf6-dependent and Rab11-dependent membrane trafficking during cytokinesis (Fielding et al., 2005; Horgan and McCaffrey, 2009). To understand the role of class II FIPs in the cortical development, we first examined the gene expression of *FIP3*, *FIP4*, and *Arf6* in the developing cerebral cortex by a nonradioactive *in situ* hybridization analysis. In the cerebral cortex at E14.5 and E16.5, *FIP3* and *Arf6* mRNAs were already detectable in the VZ and maintained throughout the cerebral cortex, while *FIP4* mRNA was expressed preferentially in the CP with a negligible level in the VZ, SVZ, and IZ (Fig. 7A). Since the expression pattern of *FIP3* traced well with that of *Arf6*, we hereafter focused on FIP3 as a candidate for Arf6 downstream effector in neuronal migration. In the immunoblot analysis of the developing cerebral cortex, a single immunoreactive band for FIP3 was already detectable at E14.5 and the expression level was gradually increased after birth (Fig. 7B). Immunofluorescent staining of the cerebral cortex at E17.5, which had been transfected with mCherry at E14.5 by *in utero* electroporation, revealed that the immunofluorescence for FIP3 appeared as numerous puncta that partially colocalized with Arf6, EEA1, and STX12 in migrating neurons in the IZ (Fig. 7C). These results suggest the occurrence of FIP3 at subpopulations of early and recycling endosomes with Arf6 in migrating neurons in the IZ.

### The Arf6-FIP3 pathway is required for neuronal migration in the IZ.

**Figure 8.**
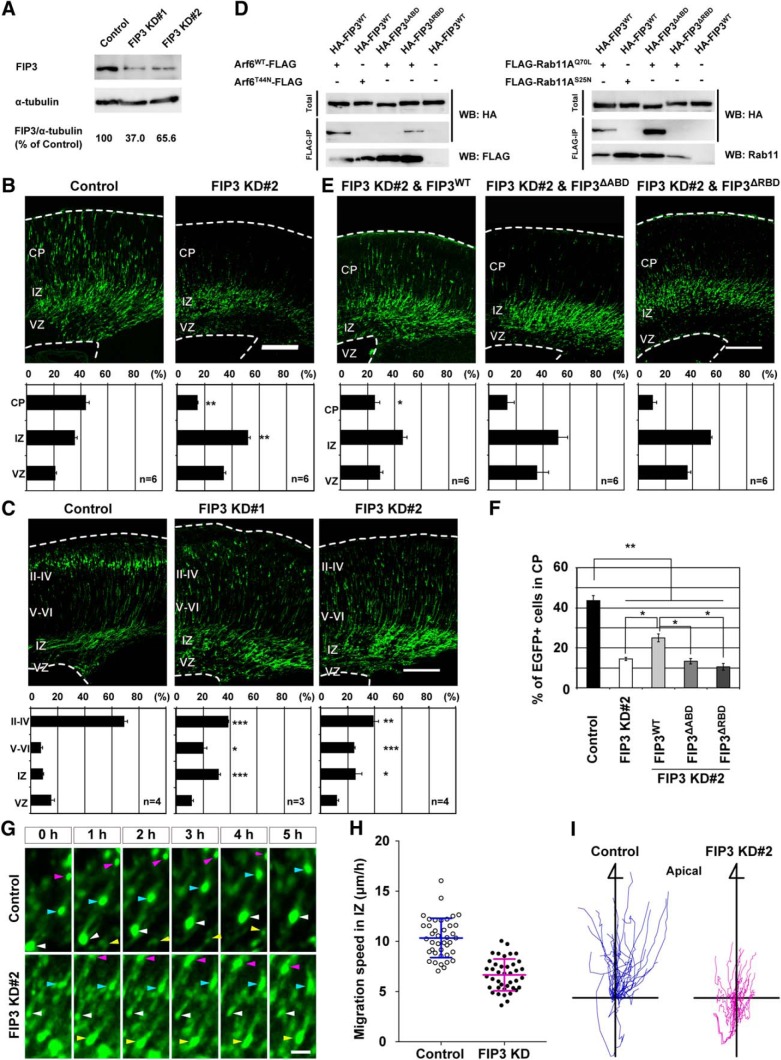
**FIP3 regulates neuronal migration in the IZ through the interaction with Arf6 and Rab11. *A***, Immunoblot analysis showing the efficiency of FIP3 shRNAs (FIP3 KD#1 and #2) in primary cortical neurons. The numbers indicate the percentage of endogenous FIP3 expression level relative to α-tubulin. Note that the expression of FIP3 KD#1 and #2 decreased endogenous FIP3 by 37.0 and 65.6%, respectively. ***B***, ***C***, ***E***, Representative images of coronal sections of E17.5 (***B***and ***E***) and P0 (***C***) cerebral cortices electroporated with Control, FIP3 knockdown (***B*** and ***C***), or FIP3 KD#2 and FIP3^WT^, FIP3^ΔABD^, or FIP3^ΔRBD^ (***E***) in combination with pCAGGS-EGFP at E14.5. The bottom graphs show the quantification of the distribution of EGFP-positive neurons in each layer. *n* in the graph indicates the number of embryos examined. ***D***, Immunoprecipitation. HeLa cells were transfected with the indicated combinations of plasmids and subjected to immunoprecipitation with anti-FLAG affinity gel. Immunoprecipitates and total lysates were subjected to an immunoblot analysis with anti-HA or anti-FLAG antibodies. Note the lack of the ability of FIP3^ΔABD^ and FIP3^ΔRBD^ to interact with Arf6 and Rab11, respectively. WB, Western blot. ***F***, Comparison of the percentage of EGFP-positive cells transfected with the indicated plasmids in the CP at E17.5. Note the ability of FIP3^WT^ but not FIP3^ΔABD^ or FIP3^ΔRBD^ to partially rescue the disturbed cortical layer formation caused by the knockdown of FIP3. ***G***, Representative time-lapse images of transfected neurons in the IZ. Embryos were electroporated with the indicated shRNAs, pCAGGS-Cre recombinase, and pCAGGS-Floxp carrying EGFP, EGFP-NLS, and EGFP-Fyn at E14.5, and subjected to time-lapse observations at E17.5. Arrowheads indicate transfected neurons. ***H***, Quantification of the migration speed of transfected neurons in the IZ (*n* = 40 cells from 3 embryos). Note the decrease in the migration speed of FIP3 KD#2-transfected neurons compared with that of control neurons. ***I***, Tracking analysis of migration of transfected neurons in the IZ. The graphs show the trajectory of migrating neurons for 10–13 h (*n* = 40 cells from 3 embryos). Data were presented as mean ± SEM (***B***, ***C***, ***E***, ***F***) and mean ± SD (***H***), and statistically analyzed using one-way ANOVA followed by *post hoc* Tukey-Kramer’s test in ***B*** (vs control) and ***E*** (vs FIP3 KD#2 in ***B***; *P < 0.05, ** *P* < 0.01), and unpaired *t* test in ***C*** and ***H***(vs control; *P < 0.05, ** *P* < 0.005, ***P < 0.0005). The total numbers of examined animals were indicated in the graphs in ***B, C,***and ***E***. Scale bars: ***B***, ***C***, ***E***, 200 μm; ***F***, 50 μm.

To determine the functional involvement of FIP3 in neuronal migration, we designed two shRNAs for FIP3 (FIP3 KD#1 and KD#2; Yazaki et al., 2014). The immunoblot analysis revealed that the expression of each of these FIP3 shRNAs effectively decreased endogenous FIP3 in cultured cortical neurons (Fig. 8A). Having established the efficiency of FIP3 shRNAs, mouse embryos were subjected to *in utero* electroporation with FIP3 shRNAs at E14.5. Three days later, the proportion of FIP3 KD#2-transfected neurons in the IZ was significantly higher than that of control neurons (Control, 34.7 ± 1.9%; FIP3 KD#2, 57.6 ± 4.0%, *P* < 0.01), and only 14.2 ± 1.0% of FIP3 KD#2-transfected neurons migrated into the CP (Fig. 8B,*F*). Disturbed cortical layer formation by the knockdown of FIP3 was still evident at P0 (Fig. 8C). FIP3 KD#1 also had the same effect on cortical layer formation as FIP3 KD#2 (Fig. 8C). In addition, the rescue experiment revealed that the cotransfection of shRNA-resistant wild-type FIP3 (FIP3^WT^) with FIP3 KD#2 partially improved the cortical migration phenotype caused by FIP3 KD#2 (Fig. 8E,*F*).

Since human FIP3 was shown to interact simultaneously with Arf6 and Rab11 through distinct binding regions in the C-terminal region (Fielding et al., 2005; Shiba et al., 2006), we prepared mouse *FIP3* mutants that lacked the ability to interact with Arf6 or Rab11 by deleting the respective binding regions [amino acids 943–1048 for Arf6 (FIP3^ΔABD^); 1069–1092 for Rab11 (FIP3^ΔRBD^)]. A coimmunoprecipitation analysis confirmed that FIP3^WT^ and FIP3^ΔRBD^, but not FIP3^ΔABD^, coprecipitated with Arf6^WT^, whereas FIP3^WT^ and FIP3^ΔABD^, but not FIP3^ΔRBD^, coprecipitated with Rab11A in a GTP-dependent manner (Fig. 8D). The cotransfection of either *FIP3^ΔABD^* or *FIP3^ΔRBD^* mutants with FIP3 KD#2 did not restore the retention of neurons transfected with FIP3 KD#2 in the IZ (Fig. 8E,*F*).

The time-lapse imaging analysis of the acute cortical slice revealed that the migration speed of FIP3-knockdown neurons in the IZ was significantly slower than that of control neurons (Fig. 8G,*H*; Control, 10.3 ± 0.3 μm/h; FIP3 KD#2, 6.7 ± 0.2 μm/h, *P* < 0.0001). Furthermore, a single-cell tracking analysis revealed that the directional migration of FIP3 KD#2-transfected neurons toward the CP in the IZ was disorganized compared with that of the control neurons (Fig. 8I). These results suggest that FIP3 regulates the neuronal migration in the IZ through the interaction with Arf6 and Rab11 during cortical layer formation.

### FIP3 regulates the trafficking N-cadherin through the interaction with Arf6 and Rab11 in migrating neurons

**Figure 9.**
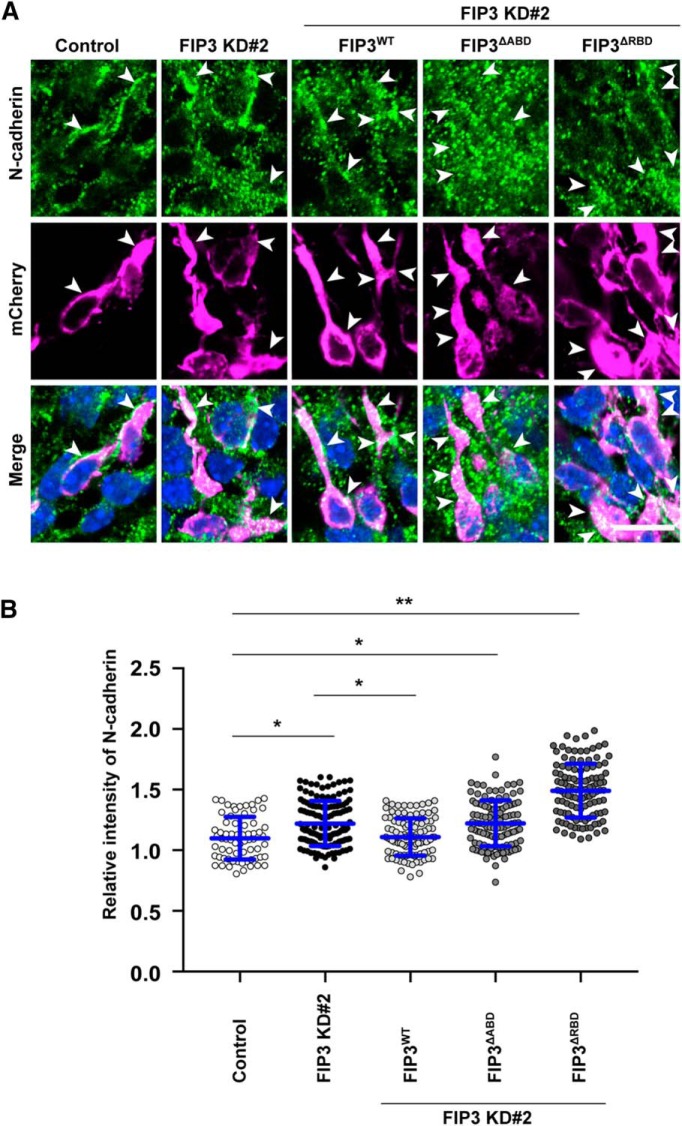
**FIP3 regulates the trafficking of N-cadherin in migrating neurons through the interaction with Arf6 and Rab11. *A***, Representative micrographs showing the effect of the expression of FIP3 shRNA with or without the indicated *FIP3* mutants on the subcellular localization of N-cadherin in migrating neurons in the IZ at E17.5. Arrowheads indicate the localization of N-cadherin in transfected neurons. ***B***, Quantification of the relative immunofluorescence intensity for cytoplasmic N-cadherin. The relative intensity was calculated by normalizing the immunofluorescence intensity for cytoplasmic N-cadherin in transfected neurons to that in the surrounding untransfected neurons. Note the cytoplasmic accumulation of N-cadherin in neurons transfected with FIP3 KD#2, which was rescued by the coexpression of FIP3^WT^, but not FIP3^ΔABD^ or FIP3^ΔRBD^ (Control, *n* = 62 cells; FIP3 KD#2, *n* = 136 cells; FIP3 KD#2 and FIP3^WT^, *n* = 96 cells; FIP3 KD#2 and FIP3^ΔABD^, *n* = 114 cells; FIP3 KD#2 and FIP3^ΔRBD^, *n* = 117 cells). Data collected from three embryos in each condition were presented as mean ± SD and statistically analyzed using one-way ANOVA followed by *post hoc* Tukey–Kramer’s test (*P < 0.05, **P < 0.01). Scale bar, 20 μm.

Finally, to determine whether FIP3 regulates the trafficking of N-cadherin downstream of Arf6 in migrating neurons, we immunohistochemically examined the subcellular localization of N-cadherin in the migrating neurons, which had been transfected with FIP3 KD#2 alone or in combination with FIP3^WT^, FIP3^ΔABD^, or FIP3^ΔRBD^. As observed in Arf6-knockdown neurons, FIP3-knockdown neurons exhibited significantly more cytoplasmic N-cadherin-immunoreactive puncta than control neurons (Fig. 9A; Control, 1.1 ± 0.2; FIP3 KD#2, 1.2 ± 0.2, *P* < 0.01). Furthermore, the cytoplasmic accumulation of N-cadherin caused by the knockdown of FIP3 was partially rescued by the cotransfection of FIP3^WT^, but not FIP3^ΔABD^ or FIP3^ΔRBD^ (Fig. 9A,*B*; FIP3 KD#2 and FIP3^WT^, 1.1 ± 0.1, *P* < 0.01 vs FIP3 KD#2; FIP3 KD#2 and FIP3^ΔABD^, 1.2 ± 0.2, *P* < 0.01 vs Control; FIP3 KD#2 and FIP3^ΔRBD^, 1.5 ± 0.2, *P* < 0.01 vs Control). These results suggest that FIP3 mediates the Arf6-dependent and Rab11-dependent trafficking of N-cadherin in migrating neurons.

## Discussion

Recent evidence implicates endosomal trafficking mediated by small GTPases, such as Rap1 and Rabs, as a critical mechanism regulating neuronal migration through the polarized delivery of membrane lipids and proteins, including N-cadherin (Kawauchi et al., 2010; Franco et al., 2011; Jossin and Cooper, 2011) and integrin (Marchetti et al., 2010; Sekine et al., 2012). Arf6 is a small GTPase that regulates a variety of neuronal functions, including neuronal migration (Falace et al., 2014), the formation of axons (Hernández-Deviez et al., 2002), dendrites, and spines (Miyazaki et al., 2005; Raemaekers et al., 2012; Kim et al., 2015), and synaptic plasticity (Scholz et al., 2010) through the endosomal trafficking and actin cytoskeleton reorganization. In this study, we provide evidence suggesting that the Arf6-FIP3 pathway regulates neuronal migration in the IZ through the endosomal trafficking of N-cadherin.

Falace et al. (2014) recently provided the first evidence for the functional involvement of Arf6 in neuronal migration and proposed that the suppression of Arf6 activation through the interaction with TBC1D24, a protein encoded by the gene associated with familiar infantile myoclonic epilepsy, is important for proper cortical neuronal migration. This conclusion was based on their findings that overexpression of a constitutively active Arf6 mutant (*Arf6^Q67L^*) but not a constitutively inactive mutant (*Arf6^T27N^*) disturbed the cortical layer formation and that the impaired neuronal migration phenotype caused by the knockdown of TBC1D24 could be rescued by the coexpression of *Arf6^T27N^*. The present study extended the previous findings by showing that overexpression of not only *Arf6^Q67L^* but also another constitutively inactive mutant (*Arf6^T44N^*) in postmitotic neurons under the NeuroD promoter disturbed cortical layer formation. The discrepancy of phenotypes caused by inactive *Arf6* mutants between previous and present studies is likely to stem from differences in experimental conditions, including inactive *Arf6* mutants (*Arf6^T27N^* vs *Arf6^T44N^*), promoters of expression vectors (chick β-actin vs NeuroD), and animal species (rat vs mouse). Concerning the biochemical properties of inactive *Arf6* mutants, Marcia et al. (2004) reported that Arf6^T44N^ is stably bound to GDP and targeted to physiologically proper membrane compartments, whereas Arf6^T27N^ tends to form aggregates in a nucleotide-free form when exogenously overexpressed *in vivo*. Thus, it is possible that Arf6^T27N^ may have given a limited dominant-negative effect on the Arf6 pathway in migrating neurons due to its improper subcellular targeting. Therefore, we propose that proper GDP/GTP cycling of Arf6 at appropriate locations and timings in migrating neurons is required for neuronal migration.

N-cadherin is known to regulate various steps in cortical development, including the maintenance of adherens junctions and polarity of radial glial progenitors in the VZ (Kadowaki et al., 2007; Gil-Sanz et al., 2014), radial glia-independent somal migration (Gil-Sanz et al., 2013), multipolar migration (Jossin and Cooper, 2011), radial glia-dependent locomotion (Kawauchi et al., 2010; Shikanai et al., 2011), neurite extension, and synaptogenesis (Bamji, 2005; Tai et al., 2007; Takeichi, 2007; Ito and Takeichi, 2009; Brigidi and Bamji, 2011). In the present study, we provided two lines of evidence suggesting the functional involvement of Arf6 in the endosomal recycling of N-cadherin to the plasma membrane in multipolar neurons. First, the knockdown of Arf6 resulted in the cytoplasmic accumulation of N-cadherin and STX12-positive recycling endosomes in migrating neurons. Second, the knockdown of Arf6 impaired the recycling of N-cadherin to the plasma membrane, but not internalization from the plasma membrane, in cultured cortical neurons. In polarized epithelial cells, there is unambiguous evidence for a functional relationship between Arf6 and E-cadherin: the activation of Arf6 facilitates the internalization of E-cadherin from adherens junctions, thereby triggering epithelial–mesenchymal transition, during wound healing and cancer invasion (Palacios et al., 2001, 2002; Luton et al., 2004). Although the directions of Arf6-mediated membrane transport are reversed between E-cadherin in epithelial cells and N-cadherin in multipolar neurons, Arf6 is suggested to affect cell motility and shape by regulating either the inward or outward transport of cadherin through the interaction with various effectors, depending on the cell type and context.

Small GTPases, such as Rap1 and Rab, are highlighted as critical regulatory pathways for surface N-cadherin expression through endosomal trafficking in neuronal migration (Kawauchi et al., 2010; Franco et al., 2011; Jossin and Cooper, 2011; Ye et al., 2014). During multipolar migration, Rap1 promotes surface N-cadherin expression in migrating neurons and their establishment of radial orientation upstream of Rac1, Cdc42, and RalA/B (Jossin and Cooper, 2011). On the other hand, Rab5 and Rab11 regulate neuronal migration through N-cadherin endocytosis and recycling (Kawauchi et al., 2010), respectively. The migratory phenotype caused by the knockdown of Arf6 is quite similar to those caused by the inhibition of Rap1 and knockdown of Rab11. Therefore, it is plausible that N-cadherin trafficking may be fine-tuned by multiple small GTPases, including Arf6, Rabs, and Rap1, in multipolar neurons along a sequential and/or parallel cascade. However, it should be noted that we failed to rescue the Arf6-knockdown phenotype of cortical layer formation by the coexpression of N-cadherin (our unpublished observations). Since either overexpression or knockdown of N-cadherin has an adverse effect on neuronal migration (Shikanai et al., 2011), our failure to rescue the Arf6-knockdown phenotype by N-cadherin may have been due to the narrow window of the optimal expression level of N-cadherin to rescue the phenotype. Alternatively, it is also possible that other cargo molecules transported with N-cadherin in endosomes are primarily responsible for Arf6-dependent neuronal migration. Therefore, a proteomic analysis to identify other cargo molecules in Arf6-positive endosomes in migrating neurons will be necessary to comprehensively understand how Arf6-dependent endosomal trafficking contributes to neuronal motility and morphology during neuronal migration.

By rescue experiments with separation-of-function *Arf6* mutants that interfere with specific downstream pathways, we identified the class II FIP family, particularly FIP3, as a possible Arf6 effector for neuronal migration in the IZ. This result was further strengthened by the following lines of evidence. First, *in situ* hybridization analysis demonstrated that the spatiotemporal expression pattern of *FIP3* rather than *FIP4* paralleled well with that of *Arf6* in the developing cerebral cortex. Further immunofluorescent analysis revealed that FIP3 partially colocalized at a subpopulation of endosomes with Arf6 and STX12 in migrating neurons. Second, the knockdown of FIP3 impaired neuronal migration in the IZ with reduced speed and disorganized directionality, which is consistent with the Arf6-knockdown phenotypes. Furthermore, the disturbed cortical layer formation caused by the knockdown of FIP3 was restored by the coexpression of shRNA-resistant wild-type FIP3, but not FIP3^ΔABD^ or FIP3^ΔRBD^. Finally, the knockdown of FIP3 also resulted in the cytoplasmic accumulation of N-cadherin in migrating neurons, similar to that of Arf6, which was restored by the coexpression of wild-type FIP3, but not FIP3^ΔABD^ or FIP3^ΔRBD^. Taken together, it is reasonable to conclude that FIP3 regulates neuronal migration downstream of Arf6 and Rab11.

Our knowledge on the physiological roles of class II FIPs largely comes from previous studies on their functional role in cytokinesis, the final stage of cell division (Fielding et al., 2005; Wilson et al., 2005; Takahashi et al., 2011; Schiel et al., 2012). FIP3 is required for the completion of cytokinesis through Arf6-dependent and Rab11-dependent endosomal transport of cargo molecules, such as p50RhoGAP and SCAMP2/3, to the midbody (Schiel et al., 2012), where FIP3-positive endosomes are tethered and fused by the interaction with the Exocyst complex via the Arf6-FIP3-Rab11 ternary complex and with the Centralspindlin complex via FIP3 (Simon et al., 2008; Takahashi et al., 2011). Arf6 also functions as an acceptor for endosomes transporting to the cleavage furrow and midbody during cytokinesis through the interaction with various components of cytokinesis, such as FIP3/4 (Fielding et al., 2005), the Sec10 subunit of the Exocyst complex (Fielding et al., 2005), mitotic kinesis-like protein MKLP1 (also known as kinesin-6 or KIF23) of the Centralspindlin complex (Makyio et al., 2012), JIP3/4 (Montagnac et al., 2009), and the serologically defined colon antigen-3 (Sakagami et al., 2016). Interestingly, recent evidence suggests that various molecular components are conserved between cytokinesis and neuronal migration. For example, the knockdown of MKLP1 or blocking of Exo70, a subunit of the Exocyst complex, was shown to result in similar disturbance of cortical layer formation to the knockdown of Arf6 (Letinic et al., 2009; Falnikar et al., 2013). Therefore, by analogy to the molecular mechanism by which the Arf6-FIP3 pathway regulates cytokinesis, we hypothesize that the activation of Arf6 may facilitate the tethering and docking of Rab11/FIP3 endosomes containing N-cadherin at the leading edge of multipolar neurons through the interaction with the Exocyst complex and MKLP1, thereby establishing the neuronal polarity and interaction with radial glial processes during the multipolar-to-bipolar transition.

In summary, the present study demonstrates that Arf6 regulates neuronal migration in the IZ through the FIP3-dependent trafficking of endosomes containing N-cadherin during cortical layer formation. The switching of Arf6 between GDP-bound and GTP-bound states is precisely regulated by multiple guanine nucleotide exchange factors and GTPase-activating proteins, which are abundantly expressed in the brain and possess multiple functional domains to interact with various proteins and lipids (Gillingham and Munro, 2007; Sakagami et al., 2008; Donaldson and Jackson, 2011). Further studies will be required to determine how the GDP/GTP cycling of Arf6 is fine-tuned in migrating neurons by these upstream regulatory factors in response to external and internal stimuli.

## Synthesis

The decision was a result of the Reviewing Editor Darcy Kelley and the peer reviewers coming together and discussing their recommendations until a consensus was reached. A fact-based synthesis statement explaining their decision and outlining what is needed to prepare a revision is listed below. The following reviewers agreed to reveal their identity: Jonathan Cooper, Jean-Bernard Manent. Through utilizing a combination of knockdown and overexpression constructs, and a variety of technical approaches, the authors quite convincingly show that Arf6 regulates multipolar migration via FIP3-dependent endosome trafficking of N-Cadherin. These are interesting novel findings that further expanded the current knowledge on the molecular pathways controlling neocortical histogenesis.

Both reviewers agree that the manuscript is now acceptable for publication, pending some very minor revision (Reviewer #2).

Reviewer #1:

I thank the authors for the revisions and am satisfied that the results are statistically significant. For the authors' information, regarding Fig 5 D, E, I questioned the significance of the internalization/recycling assay not because of the number of sample groups (n=2) but because of the number of time points (n=4). Unless there is a clear hypothesis before an experiment is done, there may be an unconscious bias to select time points at which there is a statistically significant effect and ignore the time points at which there is no effect, essentially "cherry picking" the data to get P(more than symbol) 0.05. However, since FIP3 has a known function in the exocyst it is reasonable to focus on the 45' and 60' time points. For this reason I agree with the authors that the results show a role for Arf6 in recycling internalized N cadherin back to the surface.

Reviewer #2

In this revised version, the authors have addressed most of the concerns, and their manuscript is very nicely improved. However, one minor concern regarding cell proliferation and differentiation analyses remains to be addressed. Although the authors claim that they found no difference in terms of number or position of BrdU+ cells (pg 17, ln20), it appears that this is only 'qualitative' since no quantification is provided. PSA-NCAM and Cux1 immunostainings for investigating any differentiation defects were not quantified either. The authors may wish to include quantifications, or at least to mention how many sections and animals showing no 'qualitative' difference were examined.

Also, minor cosmetic changes for improving figure clarity could be made: 1) Tracing cell contours in the highlighted cell in Fig 4B, 7C ; 2) Improving dot plots in Fig9B in order to make dots smaller and lighter (mean and SEM could hardly be seen in the present version of the fig).

Last, a few typos could be found: 'Samll GTPases' pg29, ln13; 'knockdwon of FIP3' pg31, ln2; 'ADDIN EN.CITE' pg32, ln17.
